# Oral cancer cells may rewire alternative metabolic pathways to survive from siRNA silencing of metabolic enzymes

**DOI:** 10.1186/1471-2407-14-223

**Published:** 2014-03-25

**Authors:** Min Zhang, Yang D Chai, Jeffrey Brumbaugh, Xiaojun Liu, Ramin Rabii, Sizhe Feng, Kaori Misuno, Diana Messadi, Shen Hu

**Affiliations:** 1 UCLA School of Dentistry, 10083 Le Conte Ave, Los Angeles, CA 90095-1668, USA; 2 UCLA Jonsson Comprehensive Cancer Center, 10083 Le Conte Ave, Los Angeles, CA 90095-1668, USA

## Abstract

**Background:**

Cancer cells may undergo metabolic adaptations that support their growth as well as drug resistance properties. The purpose of this study is to test if oral cancer cells can overcome the metabolic defects introduced by using small interfering RNA (siRNA) to knock down their expression of important metabolic enzymes.

**Methods:**

UM1 and UM2 oral cancer cells were transfected with siRNA to transketolase (TKT) or siRNA to adenylate kinase (AK2), and Western blotting was used to confirm the knockdown. Cellular uptake of glucose and glutamine and production of lactate were compared between the cancer cells with either TKT or AK2 knockdown and those transfected with control siRNA. Statistical analysis was performed with student T-test.

**Results:**

Despite the defect in the pentose phosphate pathway caused by siRNA knockdown of TKT, the survived UM1 or UM2 cells utilized more glucose and glutamine and secreted a significantly higher amount of lactate than the cells transferred with control siRNA. We also demonstrated that siRNA knockdown of AK2 constrained the proliferation of UM1 and UM2 cells but similarly led to an increased uptake of glucose/glutamine and production of lactate by the UM1 or UM2 cells survived from siRNA silencing of AK2.

**Conclusions:**

Our results indicate that the metabolic defects introduced by siRNA silencing of metabolic enzymes TKT or AK2 may be compensated by alternative feedback metabolic mechanisms, suggesting that cancer cells may overcome single defective pathways through secondary metabolic network adaptations. The highly robust nature of oral cancer cell metabolism implies that a systematic medical approach targeting multiple metabolic pathways may be needed to accomplish the continued improvement of cancer treatment.

## Background

The pentose phosphate pathway (PPP) is a biological process that mainly functions to produce ribose-5-phosphate for nucleic acid synthesis and to generate nicotinamide adenine dinucleotide phosphate (NADPH) [1]. There are two distinct branches of the pathway: the oxidative PPP that converts glucose-6-phosphate into pentose phosphate metabolites, and the non-oxidative PPP that recycles pentose phosphates to glycolytic intermediates or generates de novo ribose-5-phosphate from glycolytic intermediates. Transketolase (TKT) is one of the rate-limiting enzymes in the PPP. Together with transaldolase, TKT converts D-pentose (xylulose and ribose) 5-phosphate into D-glyceraldehyde 3-phosphate and D-fructose 6-phosphate, and TKT also utilizes these glycolytic intermediates for de novo synthesis of ribose-5-phosphate in the non-oxidative phase of PPP. In cancer cells, the PPP catalyzed by TKT plays an important role in utilizing glucose for ribose-5-phosphate synthesis [2]. Ribose-5-phosphate can be synthesized from the glycolytic intermediates, fructose-6-phosphate and glyceraldehyde-3-phosphate, via the non-oxidative branch of PPP or from glucose-6-phosphate via the oxidative branch of PPP. Previous studies have underlined the importance of TKT for tumor cell metabolism, by demonstrating that enhancement of TKT activity supports tumor cell survival and proliferation [3]. In 2005, a transketolase-like protein 1 (TKTL1) was identified as a possible mutant form of human TKT [4]. The protein was found to be over-expressed in multiple types of cancer tissues [5-7] and contribute to a malignant phenotype through increased glucose metabolism even in the presence of oxygen and stabilization of hypoxia-inducible factor 1-alpha (HIF-1α) [8]. Inhibition of TKTL1 gene expression in tumor cells resulted in decreased cell growth and proliferation as well as reduced glucose metabolism and lactate production [9]. In addition, TKTL1 is indispensable for the function of the p53-dependent effector TIGAR (Tp53-induced glycolysis and apoptosis regulator) on hypoxia-induced cell death [10], and its expression correlates with HIF-1α expression and is induced upon hypoxic conditions which facilitate energy supply to tumors under these circumstances [10]. These findings have demonstrated TKTL1 may play an important role in the pathophysiology of malignant tumors.

Adenylate kinases (AKs) represent a set of enzymes that catalyze a reversible high-energy phosphoryl transfer reaction between adenine nucleotides [11]. So far, six AK isozymes, AK1, AK2, AK3, AK4, AK5, and AK6, were identified. AK1 is localized in neuronal processes, sperm tail and on the cytoskeleton in cardiac cells at high concentrations whereas AK2 is expressed in the intermembrane space, and AK3 and AK4 are localized in the mitochondrial matrix. AK3 is expressed in all tissues except for red blood cells suggesting that AK3 gene is a housekeeping-type gene. However, AK4 is tissue-specific, mainly expressed in kidney, brain, heart, and liver while AK5 is solely expressed in a limited area of brain [12-14]. AK2 is a crucial component of this AK relay mechanism, unique in its localization, and it functions to maintain low cytosolic AMP concentrations as it primarily utilizes and sequesters AMP [11]. During periods of metabolic stress, AK2 increases the amount of available AMP and therefore the AMP:ATP ratio, which activates downstream ATP-sensing mechanisms – such as AMP-activated protein kinase (AMPK) – to regulate cellular metabolism. AK2 gene mutation has been found in patients with reticular dysgenesis, a most severe form of SCID (human severe combined immunodeficiencies) which may lead to bilateral sensorineural deafness in affected newborns. AK2 is specifically expressed in the stria vascularis region of the inner ear, providing an explanation of the sensorineural deafness in these individuals [15,16]. AK2 is also essential for the development of Drosophila melanogaster [17] as well as in the process of cardiac stem cell differentiation/cardiogenesis. Knockdown of AK1, AK2 and AK5 activities with siRNA disrupted cardiogenesis whereas induction of creatine kinase, the alternate phosphotransfer pathway, compensated for AK-dependent energetic deficit [18]. In addition, human immunodeficiency virus type 1 (HIV-1) encoded viral protein Vpr was found to induce the expression of enzymes in the glycolytic pathway (pentose phosphate and pyruvate metabolism) whereas down-regulate AK2 and TKT [19]. While AK2 is a significant component of cellular metabolism and adaption, it has not yet been linked to oral cancer. Previous studies have demonstrated a biochemical difference of AK2 in lung tumors, but evidence of its role in other types of cancers is limited [20,21].

The purpose of this study is to test if oral cancer cells can overcome the defects introduced by using small interfering RNA (siRNA) to knock down important metabolic enzymes. siRNA silencing of TKT or AK2 was performed on UM1 and UM2 oral cancer cells, which were both derived from a same tongue squamous cell carcinoma, and the effects of their inhibition on cell proliferation, cellular uptake of glucose and glutamine and lactate production were investigated.

## Methods

### Cell lines and cell culture

Normal human oral keratinocytes (NHOKs) were maintained in EpiLife media supplemented with the human keratinocyte growth supplement (Invitrogen, Carlsbad, CA, USA). UM1 and UM2 oral cancer cell lines [22] were cultured in Dulbecco’s modified eagle medium (DMEM) plus 10% fetal bovine serum, penicillin (100 U/mL), and streptomycin (100 μg/mL). The cells were maintained at 37°C in a humidified 5% CO_2_ incubator and passaged when they reached 90-95% confluence.

### siRNA knockdown of TKT or AK2

UM1 and UM2 cells were transfected with siRNA in 96-well or 6-well plates using the Hilymax transfection regent (HilyMax, Rockville, MD, USA) according to the company’s instruction. Validated double-stranded siRNAs of TKT (sc-45591) and AK2 (sc-38906) or non-target control siRNAs (Santa Cruz Biotech, Santa Cruz, CA, USA) were mixed with the transfection reagent and then added to the cell culture. After 24-hour treatment, the siRNAs were removed and the cells were further cultured in fresh media for 48 hours. Both siRNAs are a pool of three different siRNA duplexes. TKT siRNA (sc-45591A): Sense: CCUACACCGGCAAAUACUUtt, Antisense: AAGUAUUUGCCGGUGUAGGtt; TKT siRNA (sc-45591B): Sense: GCAUCUAUAAGCUGGACAAtt; Antisense: UUGUCCAGCUUAUAGAUGCtt; TKT siRNA (sc-45591C): Sense: CUUCUCGGAGAUCUUCAAAtt; Antisense: UUUGAAGAUCUCCGAGAAGtt. AK2 siRNA (sc-38906A): Sense: GAAGCUUGAUUCUGUGAUUtt; Antisense: AAUCACAGAAUCAAGCUUCtt; AK2 siRNA (sc-38906B): Sense: CCAGCCAGUUAGUUAUUCAtt; Antisense: UGAAUAACUAACUGGCUGGtt; AK2 siRNA (sc-38906C): Sense: GCACUUGCUUGAUGUAUCUtt; Antisense: AGAUACAUCAAGCAAGUGCtt.

### Cell proliferation assay

Cell proliferation assays were performed at 72 hours post-transfection using the CellTiter 96® aqueous non-radioactive MTT cell proliferation kits (Promega, Madison, WI, USA). Briefly, 20ul of the MTT/PMS mix was added to each well of the 96-well plate and incubated at 37°C in CO_2_ incubator for 60 min. The absorbance was read at 490 nm with a 96-well plate reader (BioTek Instruments, Winooski, VT, USA). Cell numbers were counted at 72 hours post-transfection using trypan-blue exclusion assay.

### Glucose assay

UM1 and UM2 cells were transfected with TKT siRNA (siTKT), AK2 siRNA (siAK2) or control siRNA (siCTRL) as described above. At 72 hours post-transfection, the transfected cells were incubated with complete culture media, and the concentration of the remaining glucose in the culture media was measured with a glucose assay kit (BioVision, Mountain View, CA, USA). Briefly, the media ware de-proteinized with a 10-KDa ultracentrifuge filter unit (Millipore, Billerica, MA, USA). The media were then diluted with phosphate buffer saline and subjected to enzymatic reactions at 37°C for 30 min in the dark, and then the absorbance was read at 570 nm with the 96-well plate reader (BioTek).

### Lactate assay

Similarly, the culture media for siRNA-transfected cells were harvested for lactate assay. The lactate in the culture media was measured with a colorimetric lactate assay kit (BioVision). The reactions were incubated at 37°C for 30 min in the dark, and then the absorbance was read at 570 nm with the 96-well plate reader.

### Glutamine assay

The remaining levels of glutamine in the culture media were measured with a glutamine assay kit (BioAssay Systems, Hayward, CA, USA). The culture media were first de-proteinized with a 10-KDa ultracentrifuge filter unit, and then incubated with the enzyme mix at room temperature for 40 min in the dark. The absorbance was read at 565 nm with the 96-well plate reader.

### Western blotting

Protein samples were separated with a 4-12% Bis-Tris NuPAGE gel (Invitrogen, Carlsbad, CA, USA) and transferred onto nitrocellulose membrane by Trans-blot SD semi-dry transfer cell (Bio-Rad, Hercules, CA, USA). The membrane was then blocked with 5% non-fat dry milk (Santa Cruz, USA) for one hour, and then sequentially incubated with anti-TKT (H50, sc-67120) or anti-AK2 (H65, sc-28786) primary antibody (Santa Cruz Biotech, Santa Cruz, CA, USA) and corresponding secondary antibody (GE Healthcare, Piscataway, NJ, USA). The detection was performed with the ECL-Plus Western blotting reagent kit (GE Healthcare).

## Results

### Knockdown of TKT or AK2 expression in UM1 and UM2 oral cancer cells

**Figure 1:**
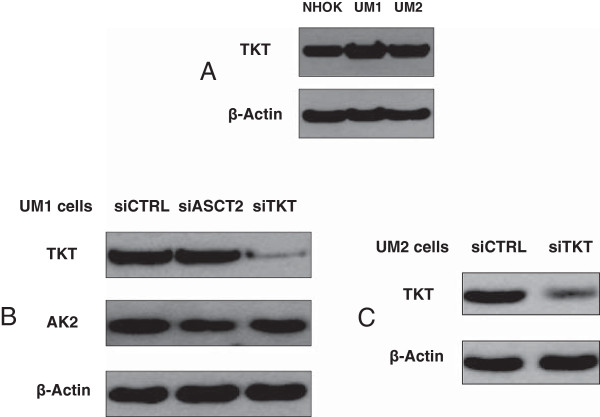
**Knockdown of TKT expression in UM1 and UM2 oral cancer cells with siRNA. (A)** Western blot analysis of TKT protein expression in NHOKs, UM1 and UM2 oral cancer cells. **(B)** Western blot analysis of TKT and AK2 expression in UM1 cells transfected with control siRNA (siCTRL), TKT siRNA (siTKT) or ASCT2 siRNA (siASCT2). **(C)** Western blot analysis of TKT in UM2 cells transfected with siTKT or siCTRL.

To investigate if TKT is abnormally expressed in oral cancer cells, we compared the endogenous expression levels of TKT among NHOKs, UM1 and UM2 cell lines. Both UM1 and UM2 cancer cell lines were initially established from a same tongue squamous cell carcinoma. However, UM1 cells appear to be highly invasive whereas UM2 cells are low invasive [22]. Western blot analysis indicated that TKT expression was slightly over-expressed in the UM1 and UM2 cancer cells when compared to NHOKs (Figure 1A). In order to assess the effects of TKT down-regulation on cellular uptake of glucose and glutamine, we used siRNA to knock down TKT expression in the UM1 and UM2 cells. As shown in Figure 1, siTKT transfection led to a significant decline in the expression of TKT in both UM1 and UM2 cancer cells.

**Figure 2:**
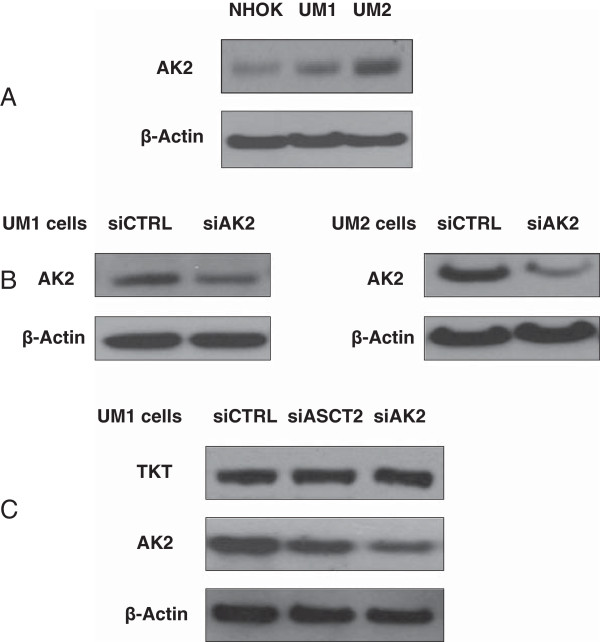
**siRNA knockdown of AK2 expression in UM1 and UM2 oral cancer cells. (A)** Western blot analysis of AK2 protein expression in NHOKs, UM1 and UM2 oral cancer cells. **(B)** Western blot analysis of AK2 expression in UM1 and UM2 cells transfected with siCTRL or siTKT. **(C)** Western blot analysis of AK2 and TKT in UM1 cells transfected with siCTRL, siAK2 or siASCT2.

AK2 was over-expressed in the oral cancer cells, especially in low invasive UM2 cells, when compared to NHOKs (Figure 2A). With siAK2 transfection, the expression of AK2 was successfully inhibited in both UM1 and UM2 cells (Figure 2B). It was also demonstrated that knockdown of TKT did not affect AK2 expression, and conversely knockdown of AK2 did not impact the expression of TKT. However, knockdown of ASCT2, which is a glutamine transporter, suppresses the expression of AK2 in UM1 cells (Figure 2C). siRNA silencing was also attempted to knock down the expression of TKT and AK2 in NHOKs. However, the normal keratinocytes did not survive from the siRNA transfection experiments.

### Effect of TKT or AK2 knockdown on the proliferation of UM1 and UM2 cells

**Figure 3:**
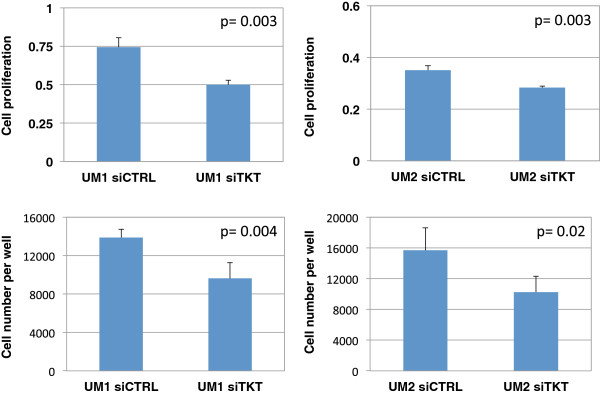
**Effect of siRNA knockdown of TKT on the proliferation of UM1 and UM2 cells.** siRNA silencing of TKT significantly inhibited the proliferation of UM1 cells (n = 4) and UM2 cells (n = 4).

To study the effect of down-regulation of TKT or AK2 on cell proliferation, UM1 and UM2 cells were transfected with either siTKT (or siAK2) or scrambled siCTRL and then harvested at 72 hours post-transfection for cell proliferation assays. As shown in Figure 3, the siTKT-transfected UM1 cells displayed a 33% reduction in cell proliferation (MTT assay) when compared to the cells transfected with siCTRL (p = 0.003), and the UM2 cells exhibited a 23% reduction in proliferation (p = 0.003). As for cell viability (trypan-blue exclusion) assays, a reduction of 30% was observed in siTKT-transfected UM1 cells (p = 0.004) and a reduction of 35% was observed in siTKT-transfected UM2 cells (p = 0.02).

**Figure 4:**
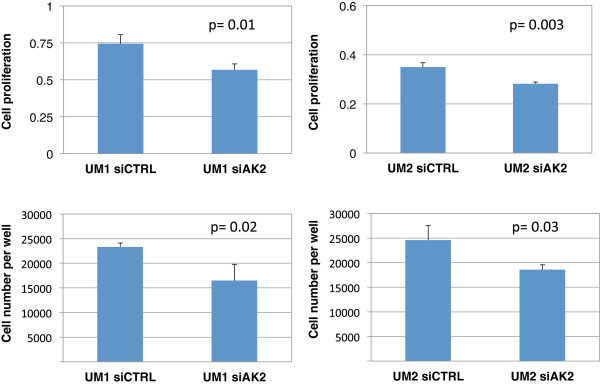
**Effect of siRNA knockdown of AK2 on the proliferation of UM1 and UM2 cells.** siRNA knockdown of AK2 significantly inhibited the proliferation of UM1 (n = 4) and UM2 (n = 4) cells.

The proliferation of UM1 and UM2 cells was also significantly inhibited when the cells were transfected with siAK2. When compared to siCTRL-transfected cells, siAK2-transfected UM1 cells exhibited a 23% decrease of proliferation (p = 0.01), while siAK2-transfected UM2 cells showed a 24% decline in their proliferation (p = 0.003) (Figure 4). Similarly, significant reductions in viable cell numbers (trypan-blue exclusion assay) were observed in siAK2-transfected UM1 (29%, p = 0.02) and UM2 cells (25%, p = 0.03).

### Effect of TKT knockdown on the glucose uptake and lactate production by UM1 and UM2 cells

To investigate the effect of TKT and AK2 knockdown on the glucose uptake of UM1 and UM2 cells, we transfected the cells with siTKT, siAK2 or siCTRL and then culture the siRNA-transfected cells in the complete growth media. Afterwards, the culture media were collected for glucose and lactate assays to determine the glucose uptake as well as the lactate production.

**Figure 5:**
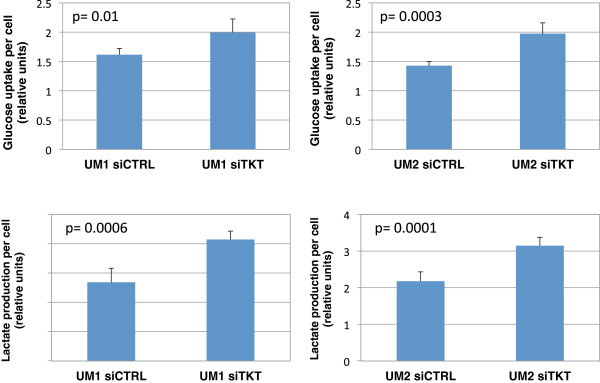
**Effect of siRNA knockdown of TKT on glucose uptake and lactate production.** A significantly higher amount of glucose was consumed by UM1 (n = 5, p = 0.01) and UM2 (n = 5, p = 0.0003) cells when TKT expression was inhibited by siRNA silencing. Meanwhile, both siTKT-transfected UM1 (n = 5, p = 0.0006) and UM2 cells (n = 5, p = 0.0001) produced significantly higher amount of lactate than the siCTRL-transfected cells.

As shown in Figure 5, siTKT-transfected UM1 cells consumed 1.24-fold higher amount of glucose (p = 0.01) than the cells transfected with scrambled siCTRL, while UM2 cells utilized 1.39-fold higher levels of glucose (p = 0.003). Meanwhile, siTKT-transfected UM1 cells produced 1.54-fold more of lactate (p = 0.0006) whereas siTKT-transfected UM2 cells produced 1.44-fold more of lactate (p = 0.0001), which corroborates with the observation of increased glucose uptake by siTKT-transfected cells.

### Effect of AK2 knockdown on the glucose uptake and lactate production by UM1/UM2 cells

**Figure 6:**
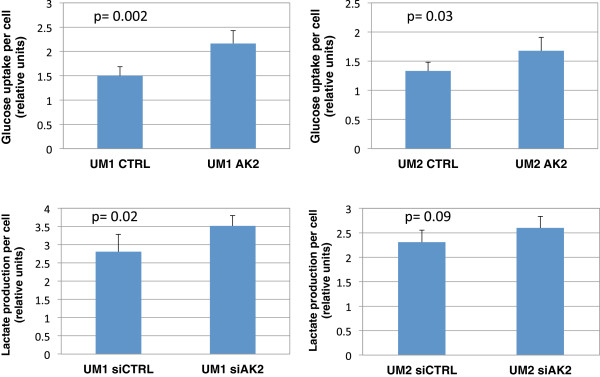
**Effect of AK2 knockdown on the glucose uptake and lactate production by UM1 and UM2 cells.** A significantly higher amount of glucose was consumed by UM1 cells (n = 5, p = 0.002) and UM2 cells (n = 5, p = 0.03) when cellular AK2 expression was inhibited by siRNA. Meanwhile, siAK2-transfected UM1 (n = 5, p = 0.02) and UM2 cells (n = 5, p = 0.09) produced more lactate than the siCTRL-transfected cells.

Glucose depletion assays were also performed to determine the glucose uptake by siAK2-transfected UM1 and UM2 cells (Figure 6). The siAK2-transfected UM1 cells utilized 1.44-fold more of glucose (p = 0.002) than the UM1 cells transfected with siCTRL, and the siAK2-transfected UM2 cells consumed a 1.26-fold more of glucose (p = 0.03). Similar to the TKT-transfected UM1 cells, the AK2-transfected UM1 cells secreted a 1.25-fold higher amount of lactate (p = 0.02), consistent with the increased uptake of glucose by the cells. However, there was only a slight increase of 1.13-fold in lactate production by the AK2-transfected UM2 cells (p = 0.09).

### Effect of TKT or AK2 knockdown on the glutamine uptake by UM1 and UM2 cells

**Figure 7:**
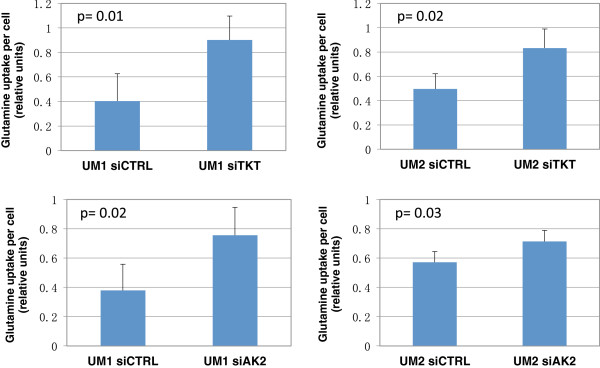
**Effect of siRNA knockdown of TKT or AK2 on the glutamine uptake by UM1 and UM2 cells.** A significantly higher amount of glutamine was consumed by siTKT-transfected UM1 (n = 4, p = 0.01) and UM2 (n = 4, p = 0.02) cells when compared to the siCTRL-transfected cells. Similarly, siAK2-transfected UM1 (n = 4, p = 0.02) and UM2 (n = 4, p = 0.03) cells consumed a significantly higher amount of glutamine than the siCTRL-transfected cells.

As shown in Figure 7, the siTKT-transfected UM1 cells utilized 2.23-fold higher amount of glutamine (p = 0.01) than the UM1 cells transfected with scrambled siCTRL, while the siTKT-transfected UM2 cells utilized 1.7-fold higher amount of glutamine (p = 0.02) than the siCTRL-transfected UM2 cells. Similarly, the siAK2-transfected UM1 cells consumed 1.99-fold more of glutamine (p = 0.02) than the siCTRL-transfected UM1 cells, whereas the siAK2-transfected UM2 cells consumed 1.25-fold more of glutamine (p = 0.03) than the UM2 cells transfected with scrambled control siRNA.

## Discussion

Tumor cell proliferation requires rapid synthesis of biomolecules such as nucleic acids, proteins and lipids. The metabolism of such tumor cells deviates from the norm, re-programmed to facilitate the uptake of nutrients such as glucose and glutamine for de novo synthesis of biomolecules. TKT, a critical metabolic enzyme in the non-oxidative branch of PPP, catalyzes reactions that enable oxygen-independent glucose degradation and that play a crucial role in the biosynthesis of ribose-5-phosphate in tumor cells. Our study demonstrates that siRNA knockdown of endogenous TKT expression significantly impairs the proliferation of the UM1 and UM2 oral cancer cells. The resultant limitation of UM1 and UM2 growth stems from the severe defects in the PPP caused by silencing TKT, affecting the biosynthesis of ribose-5-phosphate, nucleotides and possibly precursors for other biomolecules. Due to siRNA silencing of TKT in these cells, ribose-5-phosphate may have to be mainly synthesized from the glycolytic intermediate glucose-6-phosphate via the oxidative branch of PPP. However, this pathway alone is less efficient in the synthesis of ribose-5-phosphate, contributing to a reduced proliferation rate of the cells. Meanwhile, PPP involves the biosynthesis of glycolytic intermediates such as fructose-6-phosphate and glyceraldehyde-3-phosphate, which are normally recycled by glycolytic process. Silencing TKT in the cancer cells obviously abolish the cross-talk between PPP and glycolysis, causing deficiency in utilizing glycolytic metabolites for down-stream biosynthesis and energy production in the cells.

When compared to the cells transfected with scrambled control siRNA, the cells transfected with siTKT also display an increased uptake of glucose. The high level of glycolysis is required to confer metabolic adaption when facing defects in the PPP, providing not only ATP for the tumor cells’ high bioenergetic demands, but also increasingly needed precursors for the synthesis of biomolecules. Consistent with the results of increased glucose uptake and glycolysis, higher levels of lactate production were observed in the cells with TKT knockdown. When endogenous TKT expression is inhibited by siRNA, UM1 and UM2 cells also uptake a markedly higher amount of glutamine. Glutamine and its degradation products, glutamate and aspartate, are precursors for de novo synthesis of nucleotides and proteins. Our study suggests that, to survive from TKT silencing, UM1 and UM2 cells increasingly utilize glucose and glutamine to rescue the production of ribose-5-phosphate, nucleotides and other biomolecules. In addition to composing the structural units of nucleic acids, nucleotides play a central role in metabolism by serving as sources of biochemical energy, such as ATP and GTP. It is thus possible that an increase in the uptake and metabolism of glucose and glutamine contributes to maintaining the chemical energy in the cancer cells.

Recently, Zhao et al. demonstrated that inhibition of TKT led to a dramatic growth inhibition of imatinib-resistant cancer cells, but co-expression of TKTL1 in those cancer cells prevented the growth inhibitory effect of TKT suppression. When imatinib-resistant cells were first stably expressed with TKTL1 that lacks the TKT shRNA sequence, the transfection of the TKT shRNA plasmid had no effect on the ability of cells to grow in the presence of both imatinib and puromycin [23]. These results are in line with the findings from our study. Since TKTL1 activity has been linked to increased glucose uptake and lactate production in cancer cells [8-10], the increased glucose uptake and lactate production observed in our study may be due to increased TKTL1 activity to compensate knockdown of TKT.

Comparable to the results obtained with the TKT knockdown study, the siRNA knockdown of AK2 also impairs the proliferation of UM1 and UM2. Due to its unique localization in the mitochondrial intermembrane space, the crucial function of AK2 in maintaining AMP: ATP ratios cannot be compensated by other ADK isozymes. Because AK2 primarily utilizes AMP to produce ADP and eventually ATP, its knock-down will inhibit the ability of the cell to generate ATP, resulting in an increased AMP:ATP ratio. Consequently, AMPK, a kinase sensitive to AMP:ATP ratios, is activated to regulate cellular energetics and restore energy homeostasis through the phosphorylation of many downstream targets [24,25]. The overall effect of AMPK activation results in the reduced proliferation of UM1 and UM2, as it is well established that AMPK deters cell growth and down-regulates anabolic pathways, such as protein and lipid synthesis, in order to conserve ATP [26].

While AMPK acts to conserve ATP through a reduction in cell growth and proliferation, its activation also results in the up-regulation of energy-producing catabolic pathways, including glycolysis [24,26]. The UM1 and UM2 cells transfected with siAK2 display an elevated consumption of glucose and glutamine, both essential for the production of cellular energy. AMPK has been referred to as a “master switch” of metabolism and glycolysis, and it has been indicated in studies of tumor cells that AMPK highly regulates cellular glycolytic flux [24,27]. The increased levels of lactate production serve as an indicator of the increased level of glycolysis, and it has been noted that greater concentrations of lactate can further stimulate AMPK activity [27]. While AMPK has an established and well-studied role in cancer, it has not been previously linked with AK2 in this context, and recently investigated functions of AMPK in regulating cell energy metabolism and tumor cell survival are still emerging [28].

## Conclusion

**Figure 8:**
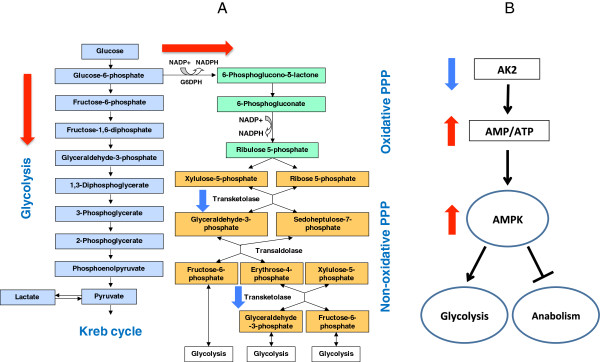
**A schematic diagram of possible metabolic network adaptations in survived UM1 and UM2 oral cancer cells after siRNA silencing of TKT or AK2. (A)** siRNA silencing of TKT may possibly result in an increased level of glycolysis and oxidative PPP in survived oral cancer cells. Compensation of TKT by TKTL1 may be involved in this metabolic adaptation because TKTL1 compensates the activity of TKT and is linked to increased glucose uptake and lactate production in cancer cells. **(B)** siRNA silencing of AK2 may lead to high AMP:ATP ratio, possibly activating AMPK and glycolysis.

In this study, we have demonstrated that the knockdown of TKT or AK2 expression significantly inhibits the proliferation of oral cancer cells. Due to deficiencies in the PPP caused by siRNA silencing of TKT, UM1 and UM2 cells displayed an increasing uptake of glucose and glutamine, and consequently produced a higher amount of lactate. These findings suggest that the partial inactivation of the non-oxidative PPP due to siRNA silencing of TKT was compensated by the activation of alternative modes of metabolism, possibly including glycolysis, TKTL1-mediated non-oxidative PPP and oxidative PPP (Figure 8). With the siRNA silencing of AK2, depletion of ATP and an increase of the AMP:ATP ratio may activate AMPK as a master regulatory metabolic control, both down-regulating anabolic pathways to preserve ATP and up-regulating catabolic mechanisms to generate ATP. Thus, the defects in the ATP-producing capacity and phosphoryl relay mechanism of AK2 may be compensated by alternative metabolic pathways initiated by AMPK (Figure 8). The implication of our study is that cancer cells, as a system, can rewire alternative pathways for biosynthesis when one of the biosynthetic pathways is truncated or suppressed. The highly robust nature of cancer cell metabolism can logically complicate cancer treatment since therapeutic intervention targeting one of the metabolic pathways cannot prevent cancer cells from utilizing other pathways for biosynthesis and proliferation. As such, a more comprehensive, systematic medical approach targeting multiple metabolic pathways may be needed to accomplish the continued improvement of cancer treatment.

## Competing interests

The authors declare that they have no competing interests.

## Authors’ contributions

MZ, YC, RR, SF and KM carried out cell culture, siRNA knockdown studies, Western blot analysis and cellular assays. SH, MZ, XL, DM conceived of the study and participated in its design. YC, JB and SH performed statistical analysis and interpreted the results. JB, DM and SH drafted the manuscript. All authors read and approved the final manuscript.

## Pre-publication history

The pre-publication history for this paper can be accessed here:

http://www.biomedcentral.com/1471-2407/14/223/prepub
